# Pharmaceutical Cocrystals in Drug-Delivery Technologies: Advances from Rational Design to Therapeutic Applications

**DOI:** 10.3390/pharmaceutics18010128

**Published:** 2026-01-20

**Authors:** Marina Monserrat Marcos Valdez, Norma Rebeca Sperandeo, Maria Soledad Bueno, Claudia Garnero

**Affiliations:** Departamento de Ciencias Farmacéuticas, Facultad de Ciencias Químicas, Universidad Nacional de Córdoba, Unidad de Investigación y Desarrollo en Tecnología Farmacéutica (UNITEFA) CONICET-UNC, Córdoba X5000HUA, Argentina; marina.marcos.valdez@unc.edu.ar (M.M.M.V.); norma.sperandeo@unc.edu.ar (N.R.S.)

**Keywords:** crystal engineering, advanced formulations, emerging drug-delivery technologies, clinical translation, cocrystals

## Abstract

Pharmaceutical cocrystals are a well-established class of solid-state forms that can modulate the solubility, dissolution, stability, and bioavailability of active pharmaceutical ingredients without altering their molecular identity. Although traditional oral formulations have demonstrated translational potential, recent research has emphasized the importance of integrating cocrystals into emerging drug-delivery technologies. This review systematically analyzes recent advances in conventional and innovative cocrystal-based platforms, critically evaluating their therapeutic relevance. A comprehensive literature search was conducted, focusing on publications from the last decade, with emphasis on studies from 2020 to 2025, including peer-reviewed articles, patents, and regulatory documents. Evidence was organized into traditional oral, inhalable, intranasal, and transdermal formulations, followed by emerging platforms such as 3D printing, nano-cocrystals, and microneedles. Case studies and preclinical/clinical data were critically assessed to identify strengths, limitations, and future directions. Advancements in formulation science and novel delivery technologies are allowing pharmaceutical cocrystals to transition from laboratory innovations to clinical applications. Despite challenges in scalability, stability, and regulatory clarity, the application of cocrystals into emerging platforms highlights their potential as transformative tools in next-generation therapeutics.

## 1. Introduction

Nowadays, the term ‘pharmaceutical cocrystal’ hardly needs an introduction. After more than two decades of extensive research, pharmaceutical cocrystals have become a well-established class of solid form, recognized for their ability to modulate the physicochemical and biopharmaceutical properties of active pharmaceutical ingredients (APIs) without altering their molecular identity. Nevertheless, for clarity and consistency, the most widely accepted definition describes cocrystals as neutral, single-phase crystalline solids composed of two or more different molecular and/or ionic compounds in a definite stoichiometric ratio, which are neither solvates nor simple salts [1]. When at least one of the components is an API, and the other is pharmaceutically acceptable, the multicomponent solid is recognized as a pharmaceutical cocrystal [2,3,4].

In recent years, the study of pharmaceutical cocrystals has become a prominent topic in crystal engineering and a key component of solid-state pharmaceutical research. These multicomponent crystalline materials have demonstrated remarkable versatility in addressing long-standing challenges in drug development. By forming predictable supramolecular assemblies between APIs and suitable coformers, cocrystals enable the fine-tuning of key solid-state properties such as solubility, dissolution rate, stability, mechanical strength, hygroscopicity, compressibility, and even sensory attributes [5,6,7,8].

Several comprehensive reviews on crystal engineering developments have consolidated the accumulated knowledge on the design and development of pharmaceutical cocrystals. This includes important stages, such as prediction based on coformer selection, preparation, and structural and pharmacokinetic characterization during preformulation, as well as the scale-up and formulation of final products, which offer important opportunities to the pharmaceutical industry [9,10,11]. Numerous disciplines of literature address improvements in physicochemical and mechanical properties, as well as the wide variety of experimental and computational techniques available for their preparation. For example, Guo et al. [3] and Kumar et al. [12] provided detailed analyses of experimental and mechanochemical preparation methods, as well as the use of in silico tools for the rational design of pharmaceutical cocrystals, while Sakhiya et al. [13] emphasized the development of green and scalable synthesis routes with growing industrial applicability. On the other hand, Parkes et al. [14] examined the integration of experimental, knowledge-based, and computational cocrystal screening methods, underlining their emerging therapeutic and formulation potential. Numerous scientific reports have demonstrated that cocrystallization represents a versatile strategy for the optimization of pharmaceutical solids. Recent research efforts have built upon this consolidated knowledge base to further refine the design, synthesis, and characterization of pharmaceutical cocrystals. Initial empirical coformer screening approaches have evolved into rational strategies based on supramolecular synthon theory, computational modeling, and predictive algorithms [15,16,17]. In parallel, advances in green and scalable manufacturing methods for cocrystal preparation—such as mechanochemical synthesis, solvent-free processes, and continuous production systems—are providing a route towards environmentally sustainable and industrially viable manufacturing routes [18,19,20]. Furthermore, recent publications have started to address regulatory perspectives and guidelines for pharmaceutical cocrystals, indicating a shift in the research landscape from fundamental investigations toward industrial translation and commercial application. Altogether, these developments illustrate a maturing field that is progressively bridging the gap between fundamental discovery and technological innovation.

Despite such progress, the transition from discovery to commercialization remains challenging. For a cocrystal to reach the market, it must be successfully formulated into a stable, safe, and effective dosage form that is compliant with regulatory and intellectual property requirements. The real potential of cocrystals can only be realized when formulation science is developed in tandem with a robust understanding of their structural and thermodynamic properties. Consequently, current research increasingly focuses on integrating cocrystals into advanced drug-delivery systems that can fully exploit their advantages.

In this context, pharmaceutical cocrystals have expanded beyond their original development for oral solid dosage forms, giving rise to a broader range of formulation strategies. While topical and inhalable formulations represent natural extensions of conventional delivery approaches, cocrystals have also been integrated into emerging technological platforms, such as microneedles, 3D-printed tablets, and nanocarriers. These technologies not only expand the functional scope of cocrystals but also allow tailoring drug delivery to specific therapeutic needs, providing a route for personalized and multifunctional pharmaceutical systems.

In consideration of the aforementioned, this review provides a comprehensive overview of current knowledge on pharmaceutical cocrystal-based platforms and highlights emerging research directions that emphasize their therapeutic potential. After decades of investigation, cocrystals are beginning to move beyond basic laboratory research toward real clinical applications. Furthermore, the ability of these multicomponent crystalline forms to improve the physicochemical properties of APIs can be further exploited through their integration into new technological platforms.

## 2. Traditional Cocrystal-Based Formulations

Traditional pharmaceutical formulations have provided the first and most consolidated evidence of the therapeutic potential of cocrystals. By improving solubility, dissolution rate, and bioavailability, cocrystals have been successfully incorporated into several dosage forms, offering practical solutions to long-standing limitations of poorly soluble APIs. These conventional routes of administration—including oral, inhalable, intranasal, and topical or transdermal delivery—provide the basis for cocrystal research, demonstrating not only their feasibility in preclinical and clinical contexts but also their capacity for development into marketed products. At the same time, they highlight the importance of rational coformer selection and formulation design as critical determinants of therapeutic success. Table 1 summarizes some relevant cocrystals discussed in this section.

### 2.1. Oral Formulations

#### 2.1.1. From Formulation Challenges to Preclinical Advances

Oral administration remains the preferred route for drug delivery due to its convenience, safety, and patient compliance. However, a large proportion of APIs exhibit poor aqueous solubility, which limits their absorption and bioavailability. To overcome these difficulties, solid-state approaches—such as preparing amorphous dispersions, inclusion complexes, and particularly pharmaceutical cocrystals—have emerged as promising strategies. Cocrystals can enhance the dissolution rate, apparent solubility, and the oral bioavailability of APIs without altering their pharmacological identity by forming stable multicomponent crystalline systems.

Despite their promising attributes, highly soluble cocrystals often face solution-mediated phase transformation, reverting to the stable, less-soluble parent drug’s solid form during dissolution. This process can compromise the dissolution advantage and, ultimately, the therapeutic performance. For instance, the carbamazepine–nicotinamide cocrystal converts to the carbamazepine (CBZ) dihydrate during dissolution, which is a less-bioavailable form [27]. Similar behavior has been described for CBZ–saccharin and CBZ–succinic acid (SUC) cocrystals, which rapidly transform to CBZ dihydrate unless polymeric excipients, such as hydroxypropyl methyl cellulose, are used to delay the conversion [28,29]. Moreover, the CBZ–glutaric acid particles can transform more than 95% to the dihydrate within minutes under non-sink conditions, and the transformation rate can depend on crystal morphology [30,31]. Comparable behavior has also been observed for other cocrystals, such as the theophylline–nicotinamide cocrystal, which partially converts to theophylline monohydrate during dissolution [32]. In addition, Baldea et al. [21] reported a ketoconazole–fumaric acid cocrystal that displayed a 100-fold increase in solubility and marked enhancement in oral bioavailability in rats, without causing hepatic toxicity. Similarly, Jia et al. [33] demonstrated that the theophylline–baicalein cocrystal achieved a 6.4-fold increase in oral bioavailability in rats while also enhancing antioxidant and anti-inflammatory activities, underscoring the pharmacological relevance of improved solubility.

Furthermore, polymorphic drug transitions that occur during dissolution can significantly impact the stability and bioavailability of the pharmaceutical cocrystals. For instance, Tong et al. [34] reported the solvent-mediated phase transformation of the ethenzamide–saccharin cocrystal from the metastable Form II to the stable Form I, with a monotropic relationship, in isopropanol. During dissolution, this transformation proceeds through three consecutive stages: dissolution of Form II, nucleation of Form I, and subsequent growth of the stable form. Additionally, the different crystal surfaces of Form II have various capabilities to facilitate the nucleation templates for the stable form. During the dissolution process, the interactions between API–coformer as well as API–solvent, along with the drug supersaturation level, will undergo changes. Consequently, the stability of the cocrystal in solution may strongly influence the recrystallization of API polymorphs upon cocrystal dissociation. For example, curcumin cocrystals have been shown to favor the recrystallization of metastable Form III over the most stable Form I in a pH 1.2 buffer solution. However, when recrystallization takes place in a buffer solution containing ethanol, the phase purity of Form III is markedly reduced. Furthermore, weaker API–coformer interactions enable the metastable cocrystals to recrystallize metastable curcumin polymorphs under lower supersaturation conditions [35].

These findings emphasize the importance of formulation strategies to stabilize the cocrystal and preserve its dissolution advantages. Bhardwaj et al. [29] highlighted the impact of physical stability of cocrystals on preclinical formulations, and the essential role of surfactants, polymeric additives, and pH modulation to prevent reversion to the parent drug during preclinical dosing.

Based on these insights, several studies have demonstrated that rational cocrystal design can successfully translate into measurable preclinical benefits. For example, Kimoto et al. [36] reported that TAK-020–gentisic acid cocrystals achieved a 60-fold increase in the AUC0-24 (dose-corrected) compared to the immediate-release tablet in dog pharmacokinetic studies, outperforming advanced solubilization technologies, such as amorphous solid dispersions and nanocrystals. Likewise, Paulazzi et al. [22] demonstrated that the curcumin–*N*-acetylcysteine cocrystal exhibited 4-fold higher water solubility and a greater solubility under physiological simulated conditions (pH 6.8 or 0.1 M HCl), leading to a 166-fold reduction in the minimum effective dose in murine models.

Further improvements have been documented in terms of stability and safety. Shi and Li [37] showed that combining the flufenamic acid–nicotinamide cocrystal with precipitation inhibitors such as a vinylpyrrolidone/vinylacetate copolymer can maintain supersaturation and prevent phase conversion during dissolution. In addition, cocrystallization has also been shown to enhance chemical stability and enable modified-release profiles; for example, nicorandil cocrystals exhibited improved chemical stability and sustained drug release compared with the parent drug [38]. Additionally, recent studies of pharmaceutical cocrystal solution stability emphasize the crucial role of formulation variables, including polymers, surfactants, pH, and dissolution media, on maintaining phase transformation, underscoring the central role of rational formulation design in translating cocrystal advantages into robust performance [39].

In addition to the well-known advantages of improved solubility and dissolution, cocrystal engineering with hydrophobic coformers provides a novel alternative for developing extended-release formulations of highly water-soluble APIs without using polymeric matrices. A recent study presents a 2:1 isoniazid–curcumin cocrystal capable of modulating the release of the highly soluble API, providing sustained drug release of up to 48 h. Considering the extremely low aqueous solubility of curcumin, it is anticipated that isoniazid will be released from the cocrystal more slowly, as a consequence of the reduced overall solubility of the cocrystal. Under acidic conditions (pH 1.2), a substantial amount of curcumin form III precipitated and recrystallized on the surface of undissolved cocrystals after 4 h, thereby substantially further inhibiting the isoniazid release. In contrast, at pH 6.8, approximately 90% of isoniazid was released linearly in the first 18 h, with complete drug release after 24 h. In addition, this cocrystal also exhibits therapeutic value through potential synergistic effects [40].

#### 2.1.2. Clinical Translation of Oral Cocrystal Formulations

The clinical translation of pharmaceutical cocrystals has followed different development pathways, ranging from early regulatory approval of multicomponent solid forms to rationally designed cocrystals evaluated through dedicated clinical programs.

One of the earliest and most impactful examples of successful clinical translation is the API–API cocrystal sacubritil–valsartal (LCZ696), commercialized as Entresto^®^ (Novartis), which was developed for the treatment of heart failure and supported by extensive patent protection. Large phase III clinical trials demonstrated a significant reduction in cardiovascular mortality and heart-failure hospitalizations, leading to early termination of the pivotal study due to the clear clinical benefit observed. This case represents a landmark in the acceptance of multicomponent solid forms as clinically and regulatorily viable API products [41,42,43].

A second milestone in the clinical development of cocrystals is the tramadol–celecoxib cocrystal (CTC), an API–API system designed to combine analgesic APIs for improving analgesic therapy.

In a Phase I bioavailability study in healthy volunteers, a 200 mg dose of CTC (equivalent to 88 mg of tramadol and 112 mg of celecoxib) was administered. CTC produced consistently lower tramadol Cmax and reduced early exposure relative to tramadol alone, reflecting a slower uptake phase. This attenuation of peak concentrations is pharmacologically significant, given the established association between high tramadol Cmax and peak-related adverse effects. In contrast, for celecoxib, CTC generated a lower Cmax but a comparable AUC to the single-API reference while accelerating its absorption relative to the conventional capsule. However, the most critical comparison is the one with the concomitant administration of tramadol and celecoxib, which both Phase I trials identified as producing mutual absorption interference. In this context, CTC yielded higher Cmax and higher AUC values for both APIs than the concomitant treatment arm, demonstrating that the cocrystal eliminates the negative pharmacokinetic interaction observed when the two APIs are administered together [44,45].

These Phase I pharmacokinetic findings were subsequently evaluated in Phase III clinical trials (STARDOM1, STARDOM2, and a bunionectomy model), which provided large-scale evidence for the first time that the cocrystal formulation could produce clinically meaningful analgesic benefits. In these studies, CTC demonstrated superior pain relief compared to tramadol, celecoxib, or placebo. It also led to a reduction in the use of rescue medication and had a tolerability profile comparable to that of standard therapies. In STARDOM2, 200 mg of CTC administered in two divided doses achieved non-inferior analgesia relative to 100 mg of tramadol administered in four divided doses, while substantially lowering cumulative tramadol exposure. This outcome has potential relevance for minimizing opioid-related adverse events. Pooled analyses further support statistically significant pain reduction and trends towards improved safety [46,47].

Although still limited, such examples demonstrate the potential of cocrystals to progress from preclinical promise toward clinical reality. Importantly, the ertugliflozin–L-pyroglutamic acid cocrystal, commercially approved as part of a marketed oral antidiabetic formulation, provides further validation that cocrystal technology can yield stable, scalable, and regulatory-accepted drug products [48].

Evidence from the use of orally administered pharmaceutical cocrystals indicates a distinct pathway from formulation challenges to early clinical translation. As shown, cocrystal strategies have successfully enhanced dissolution, improved absorption and bioavailability, and, in some cases, achieved therapeutic benefits in humans.

### 2.2. Inhalable Formulations

The inhalation route has become an essential strategy for the management of numerous conditions, including asthma, chronic obstructive pulmonary disease, bacterial and viral respiratory infections, and diabetes. More recently, it has also been used to treat patients with SARS-CoV-2 infection (known as COVID-19). Its growing relevance is driven by several therapeutic advantages, such as targeted deposition in the lungs that allows rapid onset of action and improved efficacy [23], the achievement of a high local drug concentration while reducing systemic exposure and first-pass hepatic metabolism [49], and a lower incidence of systemic adverse effects due to the reduced doses required for clinical activity [50]. Additionally, dry powder formulations offer enhanced stability and minimal contamination risk when compared with liquid alternatives [23]. Also, they enable non-invasive pulmonary delivery of advanced therapeutics, such as monoclonal antibodies, nucleic acids, and vaccines [51].

Despite these advantages, pulmonary drug absorption remains limited by the unfavorable physicochemical properties of many APIs, including low solubility, dissolution rate, wettability, and permeability [52].

#### 2.2.1. Cocrystallization Strategies to Overcome Limitations of Inhaled Drugs

Cocrystallization offers opportunities to modulate aerosol performance and particle size, which are critical parameters for efficient pulmonary delivery [53]. By selecting low-molecular-weight coformers, such as small organic acids, it is possible to generate well-defined cocrystal-based dry powders with optimized solid-state properties, suitable aerodynamic behavior, and improved therapeutic potential [54,55]. Several recent studies have demonstrated the effective application of cocrystal engineering in the development of inhalable formulations, as detailed in the subsequent case studies.

#### 2.2.2. Case Studies of Inhalable Cocrystals

A significant demonstration of enhancing pulmonary bioavailability through cocrystal engineeringwas reported by Karashima et al. [52], who prepared itraconazole cocrystals with SUC and L-tartaric acid. When formulated as micronized powders, both cocrystals achieved markedly higher plasma concentrations after pulmonary administration in rats, showing bioavailabilities of 10.9% and 8.7%, respectively. These values correspond to 24-fold and 19-fold increases in the AUC0-8h, respectively, compared to the crystalline form of itraconazole. Such results highlight cocrystallization as a promising strategy to improve the dissolution rate, and absorption of, poorly soluble antifungal APIs for inhaled therapy.

In antiviral therapy, remdesivir (RDV)—an API characterized by an extremely low aqueous solubility and extensive first-pass metabolism—was cocrystallized with salicylic acid (SA) using liquid-assisted grinding followed by thermal annealing as an activated preparation method. The RDV–SA cocrystals displayed a 15.4-fold increase in drug release in simulated lung fluid compared with the raw RDV. Moreover, this RDV–SA formulation demonstrated safety in A549 cells, without any in vitro cytotoxicity (0.05–10 μM). The study underscores the pharmaceutical potential of cocrystal-based inhalable formulations for more effective pulmonary administration [23].

In addition to improving biopharmaceutical properties and therapeutic activity, cocrystals can also enhance pharmacodynamic responses. For example, the tegafur–syringic acid cocrystal developed by Yu et al. [56] significantly improved the solubility, dissolution, and permeability of tegafur, as well as its in vivo pharmacokinetics. This generated a synergistic antitumor effect that was superior to that of pure tegafur. Similarly, the niclosamide cocrystals prepared by spray-drying exhibited suitable characteristics for improving the efficacy against human lung cancer; these included spherical particles measuring 1–5 µm with enhanced aerodynamic performance, a 14.8-fold increase in solubility, and stronger antiproliferative activity in A549 cells, which was associated with increased autophagic flux in cancer cells [57].

More recently, Wong et al. [49] reported on the development of a favipiravir–theophylline cocrystal in the form of a carrier-free inhalable powder for use in respiratory infections. Using a Quality by Design-guided development approach and spray-drying, an optimized formulation with a median mass aerodynamic diameter of 2.93 µm and a fine particle fraction of 79.3% was achieved. This formulation is suitable for deep lung deposition, while maintaining excellent in vitro safety. This work demonstrated that cocrystallization can reduce the requirement for ultra-high oral doses of favipiravir and facilitate its repositioning for pulmonary delivery.

Overall, the evidence indicates that cocrystallization is a practical and effective method for converting poorly soluble APIs into viable inhalable products, supporting their use in the development of more efficient and targeted treatments for respiratory diseases.

### 2.3. Intranasal Formulations

Intranasal drug-delivery systems are being actively developed as a promising alternative to oral and parenteral formulations. They are becoming popular for the treatment of local and systemic nasal diseases, central nervous system (CNS) disorders, and for vaccine delivery [49]. Intranasal administration has emerged as a rapid, non-invasive, and efficient route for both systemic and CNS deliveries. Due to the rich vascularization of the nasal mucosa and the unique anatomical access provided by the olfactory and trigeminal pathways, APIs delivered intranasally can bypass gastrointestinal degradation and first-pass hepatic metabolism, resulting in a faster onset of action and more predictable pharmacokinetics [58,59,60].

These advantages have supported the approval and clinical adoption of several intranasal therapeutics. For example, intranasal fentanyl and ketamine provide rapid analgesic effects suitable for acute or breakthrough pain [61]. Sumatriptan and zolmitriptan achieve faster relief during migraine and cluster attacks due to rapid nasal absorption [62]. Midazolam and diazepam administered intranasally are effective rescue therapies for acute seizures [63]. In addition, oxytocin and intranasal melatonin have shown CNS-mediated behavioral and cognitive effects relevant to neuropsychiatric and neurodegenerative conditions [60,64].

These advantages have facilitated the regulatory approval and clinical implementation of multiple intranasal therapeutics. The wide versatility of this route provides a strong rationale for exploring solid-state engineering strategies, such as cocrystal design, to further extend its scope of application.

#### 2.3.1. Limitations of Intranasal Administration

Despite its advantages, intranasal delivery presents significant formulation challenges: limited dosing volume, mucociliary clearance, enzymatic degradation, and the need for rapid dissolution within the small aqueous layer covering the nasal mucosa. These constraints are especially problematic for APIs with poor aqueous solubility, slow dissolution rates, or limited permeability.

Cocrystal engineering provides several well-suited strategies to address these issues and opens the possibility of delivering APIs traditionally unsuitable for intranasal administration.

#### 2.3.2. Reports of Intranasal Cocrystals

Although the field is still in its early stages, two systems provide clear evidence that cocrystals can be successfully formulated for intranasal delivery. They illustrate complementary strategies to improve solubility, dissolution, permeability, device performance, and, ultimately, in vivo pharmacokinetics and CNS distribution.

The FAV–INA intranasal dry powder cocrystal, developed by Wong et al. [24], is an engineered system designed to overcome the low intrinsic solubility of FAV. Mechanochemical synthesis followed by spray freeze-drying yielded flake-like particles (~20 μm) optimized for nasal deposition. The FAV–INA cocrystals showed 3-fold higher permeation compared with crystalline FAV, over 80% deposition in a 3D nasal cast, and less than 6% lung penetration, confirming nasal targeting. Also, no cytotoxicity was observed in epithelial and neuronal cell lines. These results demonstrate that cocrystal engineering can meaningfully improve the nasal suitability of poorly soluble antiviral compounds.

Curcumin is a potent antioxidant compound with neuroprotective and anti-amyloid properties. However, its poor solubility and extremely limited oral bioavailability restrict its therapeutic use. Desai and Petravale [65] have successfully developed a curcumin–coformer A cocrystal that was incorporated in micellar nanocarriers to nasal spray formulation. The cocrystal micellar formulation exhibited more than 3-fold solubility enhancement, rapid and nearly complete release, and excellent neuro-compatibility (more than 90% viability in U87MG cells). Moreover, in vivo pharmacokinetic and brain biodistribution studies in rodent models showed that the formulation exhibited a relative bioavailability enhancement of about 4.5-fold compared to the intranasal curcumin solution. In addition, the half-life increased to 3.7 h, and the cocrystals showed significantly greater brain uptake than all control groups and no systemic or behavioral toxicity.

### 2.4. Topical, Transdermal, and Wound-Healing Applications

In the past three decades, transdermal delivery has gained prominence as an attractive alternative to oral administration. Its advantages include convenience and the avoidance of digestive discomfort and hepatic first-pass metabolism, reduced API–food interactions, and, in certain cases, a closer proximity between the application site and the therapeutic target [25,66]. Despite these benefits, the development of stable and effective transdermal formulations remains challenging [67]. Lipid-based systems have been extensively explored, particularly for poorly soluble APIs, but they are susceptible to oxidative degradation [68,69]. In this context, pharmaceutical cocrystals have recently emerged as a promising approach to enhance drug delivery through modulating solubility, stability, and skin permeation [70].

#### 2.4.1. Cocrystals of Nonsteroidal Anti-Inflammatory Drugs

Nonsteroidal Anti-Inflammatory Drugs (NSAIDs) are known to cause adverse gastrointestinal effects when administered orally, including ulceration, bleeding, and dyspeptic symptoms, making transdermal delivery a highly attractive alternative.

A representative example is meloxicam, a poorly soluble NSAID commonly used in chronic inflammatory disorders. Machado et al. [25] designed a meloxicam–SA cocrystal, utilizing SA as both a coformer and a permeation enhancer. Ex vivo studies demonstrated that the cocrystal increased the permeability coefficient from 1.38 to 2.15 × 10^−3^ cm/h in an aqueous suspension; however, this effect was reduced in non-ionic gel, where the value decreased to 0.42 × 10^−3^ cm/h. These results highlight the final dosage form’s impact on the advantages of crystallization.

More recently, cocrystals of flufenamic acid (FFA) have been developed with structurally related carboxamide coformers such as nicotinamide, picolinamide (PIC), and isonicotinamide (INA) [71]. These cocrystals were incorporated in oleaginous bases and evaluated for topical diffusion. Remarkably, the FFA–INA and FFA–PIC cocrystals showed approximately 4.3- and 3.3-fold higher cumulative rates over 6 h, respectively, compared with raw FFA in Vaseline formulations. Importantly, the study also revealed that cocrystal dissociation was substantially reduced in oily bases compared to aqueous media, emphasizing the critical role of formulation selection in preserving stability and maximizing delivery performance.

Together, these studies demonstrate that NSAID cocrystals can enhance topical bioavailability, reduce lag time, and potentially expand therapeutic applications beyond inflammation to include wound healing and tissue regeneration. The magnitude of these effects, however, is strongly influenced by the coformer, the resulting crystalline architecture, and the selected vehicle, underscoring the importance of integrated formulation strategies in the design of the transdermal drug-delivery systems.

#### 2.4.2. Antimicrobial and Antifungal Cocrystals

Cocrystal-based strategies for permeability enhancement have also been extended to the treatment of skin infections, demonstrating their potential in topical applications. For example, isoniazid–resveratrol cocrystals were developed as a potential topical therapy for cutaneous tuberculosis, aiming to increase local concentrations of isoniazid and improve treatment compliance compared to systemic administration [72].

Ketoconazole cocrystals provide further evidence of the therapeutic potential of this approach. For example, the ketoconazole–para-aminobenzoic acid cocrystal demonstrated enhanced solubility, antifungal efficacy, and an improved safety profile in BALBc mice. Notably, it exhibited reduced sensitization and a marked anti-inflammatory effect through the downregulation of pro-inflammatory cytokines [73]. On the other hand, the ketoconazole–fumaric acid cocrystal showed biocompatibility with skin cells and an absence of systemic toxicity in animal models. Molecular docking evidence also supports their pharmacological activity, reinforcing their translational potential [21].

Similarly, the betaine–SA cocrystal has been developed to enhance the bioactivity, biocompatibility, and clinical efficacy of SA. This cocrystal exhibited significantly reduced irritancy and cytotoxicity compared to SA alone, while maintaining excellent anti-inflammatory and antioxidant properties. In vitro studies have revealed enhanced skin penetration and sustained release, positioning this cocrystal as a promising candidate for topical acne treatment [74].

#### 2.4.3. Cocrystals in Wound Healing and Regenerative Applications

Since chronic infections frequently compromise wound healing, cocrystals are being explored not only for their antimicrobial potential but also for their ability to accelerate tissue repair. The process of wound repair involves multiple coordinated stages, such as inflammation, granulation, contraction, epithelialization, and scar remodeling, which can be severely impaired in chronic wounds [75,76].

A recent study reported a curcumin-based cocrystal as an innovative approach to accelerate tissue repair and minimize scar formation. This formulation improved the solubility and stability of curcumin while also providing antibacterial and regenerative benefits. This underscores the multifunctional potential of cocrystals in wound management [26]. Moreover, hydrogels incorporating cocrystal-derived photothermal antibacterial materials have shown potential as advanced dressings, displaying antimicrobial activity with enhanced wound closure [77].

## 3. Emerging Drug-Delivery Platforms for Cocrystals

Beyond conventional dosage forms, cocrystals are increasingly being explored within innovative drug-delivery platforms that aim to expand their functional scope and translational impact. Advances in technologies, such as 3D printing, nano-cocrystals, and microneedles, illustrate how supramolecular design can be combined with modern fabrication methods to achieve personalized, scalable, and multifunctional therapies. These platforms represent a new frontier in cocrystal research, where the challenge is not only to optimize physicochemical properties but also to integrate them into emerging technologies capable of reshaping pharmaceutical development.

### 3.1. 3D Printing

In recent years, novel alternatives to conventional cocrystal fabrication have emerged, such as 3D printing technologies [78]. Developing cocrystal-based formulations using 3D printing provides potential opportunities for obtaining patient-personalized dosage forms. This technique provides flexibility to adjust the dose, release profiles, and dosage form shape, something that would not have been possible using conventional manufacturing techniques [79].

Several improved 3D printing techniques have recently been developed that have attracted the interest of pharmaceutical formulators due to their ability to build layer by layer [80]. However, to produce cocrystals rapidly and continuously, these techniques require the optimization of critical process and equipment parameters [81]. Despite the considerable commercial potential of 3D printing in pharmaceutical applications, its specific combination with cocrystal formation for marketed products remains in development.

Recently, the combination of hot-melt extrusion with a 3D printing process has resulted in numerous applications of pharmaceutical cocrystals. For example, hot-melt extrusion has been employed to produce polymeric filaments containing cocrystals, which are subsequently used in fused deposition modeling additive manufacturing. This strategy has enabled the fabrication of printlets of 3D cocrystals with more efficient API release profiles. Nyavanandi et al. [82] developed 3D cocrystal printlets of hydrochlorothiazide and nicotinamide using this combination of techniques. The cocrystal extrudates were developed using Kollicoat^®^ IR and Kollidon^®^ VA64 as polymeric carriers, resulting in smooth and irregularity-free filaments suitable for the 3D printing process. The 3D cocrystal printlets exhibited improved hydrochlorothiazide solubility, which is a high melting point API, resulting in complete release profiles compared to the pure drug and non-cocrystal formulations. Therefore, the authors propose that this approach is a viable process for developing tablets using suitable excipients to formulate patient-focused dosage forms. In another study, a combination of Kollicoat^®^ IR and Kollidon^®^ VA64 was selected for the hydrophilic polymers to develop polymeric filaments of ibuprofen and nicotinamide cocrystals [83]. The authors reported an advantageous combination of materials in lowering the extrusion and 3D printing process temperatures. The resulting cocrystal printlets remained stable for 6 months and exhibited faster in vitro drug release profiles. Subsequently, Huang et al. [84] evaluated the preparation conditions for ibuprofen–nicotinamide cocrystals using hot-melt extrusion. The optimum cocrystals, which were predominantly of granular form, were then employed to fabricate tablets using fused deposition modeling–3D printing technology. These cocrystal-loaded tablets significantly enhanced the dissolution and release rate of ibuprofen. Notably, the tablets did not disintegrate; they gradually dissolved and were released from the 3D-printed matrix, suggesting that the polymeric matrix itself played a central role in the release process. Furthermore, the first-order release profile provides significant advantages in terms of controlling drug release, which the authors suggest could facilitate the rational design of dosage regimens.

Similarly, mosapride citrate cocrystals with saccharin sodium as a coformer were prepared by liquid-assisted grinding, using ethanol as solvent. These cocrystals were then incorporated into a printable hydroxypropyl methylcellulose gel, and floating tablets were fabricated using the fused deposition modeling technique with a printer filament composed of biodegradable thermoplastic polylactic acid. This material was selected for its optimal properties, which can be adjusted for floating purposes. The floating tablets, produced in different configurations, exhibited diverse release profiles, enabling modulation of drug release in the upper part of the stomach. This would provide the advantages of enhanced bioavailability together with decreased dosing frequency [85].

Another example is the cocrystal of sulfadimidine and 4-aminosalicylic acid, which was obtained using a novel 3D-printed microfluidic device coupled with a fluidized bed [78]. This device allowed mixing, coating, and drying to be performed in a single step. Additionally, it was demonstrated that using polyvinyl pyrrolidone as a binder in the aqueous phase during mixing inhibited the formation of needle-shaped crystals, resulting in the generation of spherical crystals with a higher level of purity.

Over the last decades, inkjet printing has been demonstrated to be a relevant technique for the rapid production of pharmaceutical cocrystals, eliminating dependence on surface-tension- or viscosity-modifying agents [86]. Seera and Guru Row [87] obtained metronidazole cocrystals with hydroxybenzoic acids using thermal inkjet printing. This method produced a uniform particle size distribution, and the results were both reproducible and repeatable. These cocrystals could be suitable for industrial-scale production as the method is more efficient than conventional techniques.

### 3.2. Nano-Cocrystals

Pharmaceutical nano-cocrystals are cocrystals engineered to have nanoscale dimensions [88] or multicomponent nanocrystals [89]. However, unlike nano-crystals (i.e., carrier-free, submicron, colloidal drug-delivery systems measuring approximately 100–1000 nanometers in size and containing one molecular species), pharmaceutical nano-cocrystals comprise an API and one or two coformers in a single multicomponent nanocrystal [89,90]. Thus, nano-cocrystals are innovative, advanced drug-delivery platforms that combine the advantages of standard cocrystals with the key principle of multicomponent nanocrystals, which is to reduce particle size to the nanoscale.

Due to the combination of cocrystallization and nanonization technologies, nano-cocrystals can exhibit improved physicochemical and biopharmaceutical properties—in some cases, synergistic properties—compared to multicomponent nanocrystals and cocrystals [91]. For example, the reduced particle size of nano-cocrystals modifies the intrinsic properties of the API or the APIs, in the case of API–API cocrystals, including their dissolution rate and equilibrium solubility. Like multicomponent nanocrystals, the dissolution rate of nano-cocrystals increases in comparison to raw APIs and standard cocrystals due to their large surface-area-to-volume ratio [92], which results from the decreased particle size, as indicated by the Noyes–Whitney equation [93]. Moreover, as with multicomponent nanocrystals, reducing the particle size of nano-cocrystals below 1 µm [93] enhances their equilibrium solubility. This effect is explained by the Ostwald–Freundlich equation, which shows that reducing the size of a solid particle to the nanoscale increases its solubility exponentially [94]. Therefore, nano-cocrystals are a promising strategy for improving the solubility and dissolution rate of APIs and drug candidates belonging to the Biopharmaceutics Classification System classes II and IV [92,93,95,96]. In fact, a higher solubility and/or dissolution rate than their respective cocrystals or multicomponent nanocrystals has been reported for 15 of the 26 nano-cocrystals listed in Table 2. Pharmaceutical nano-cocrystals can also improve the bioavailability and efficacy of APIs [92,93,95,96]. The enhanced bioavailability of nano-cocrystals in comparison with cocrystals, salts, suspensions, or the free API was demonstrated by Huang Y et al. [97], Pi et al. [98], and Yu et al. [99] for phenazopyridine–phthalimide, baicalein–nicotinamide, and cytarabine–uracil nano-cocrystals, respectively, as outlined in Table 2. Pharmaceutical nano-cocrystals can also reduce the cytotoxicity of individual APIs, such as lamivudine and zidovudine [100]. In addition, they have the ability to improve antitumor activity in vitro and in vivo, as shown by Mohammad et al. [101] and Yu et al. [99] for paclitaxel–disulfiran and cytarabine–uracil nano-cocrystals, respectively (Table 2). The hybrid nature of nano-cocrystals confers additional potential advantages. For example, nano-cocrystals can exhibit long-term physical stability, as reported by Mohammady et al. [102] for carvedilol–tartaric acid nano-cocrystals (Table 2). They can also improve mucoadhesivity [96] and thermal stability [92]. Another advantage of nano-cocrystals is that, like multicomponent nanocrystals and cocrystals, they can be synthesized using several methods [92,96], enabling the generation of nano-cocrystals with tailored physicochemical and biopharmaceutical properties. However, generating nano-cocrystals can be more complex than obtaining multicomponent nanocrystals due to the differences in solubility of APIs and coformers in solvents, and the phase instability of cocrystals in aqueous media [103].

Pharmaceutical nano-cocrystals can be produced using a one-step or a two-step process. In the former, the API and coformer are paired, in an appropriate medium, and nanonized with or without stabilizer/s. In the two-step process, the cocrystal is synthesized using a suitable cocrystallization technique—either solid-state or solution-based—and then nanonized. As shown in Table 2, the one-step process was used to generate fourteen nano-cocrystals, while the two-step process was used for twelve. On the other hand, nanonization can be carried out using a number of techniques, which fall into two categories: top-down and bottom-up approaches [88,91,92,96]. Top-down techniques utilize shear forces to reduce the particle size from the microscale to the nanoscale [88,91,96]. This process is typically carried out using wet milling, grinding (either neat or liquid-assisted grinding), or high-pressure homogenization [92,95,96]. These techniques have been successfully used for generating many of the nano-cocrystals reported in Table 2. In bottom-up approaches, nanostructures are fabricated by building up smaller nanoscale structures, such as atoms or molecules, from a solution through nucleation and crystal growth in the presence of stabilizer/s [96,104]. Stabilizers, such as polymers and surfactants, prevent agglomeration, thereby ensuring that the obtained nano-cocrystals maintain their nanoscale dimensions [92,96,100]. Common bottom-up techniques used to prepare pharmaceutical nanocrystals include antisolvent precipitation (with or without ultrasonication), solvent evaporation, cooling crystallization, sonoprecipitation, electrospraying, and spray drying [92,95,105], as shown in Table 2.

**Table 2:** A summary of the preparation methods, particle sizes, and properties of the nano-cocrystals reported in the open literature from 2016 to 2025.

Nano-Cocrystal	Method of Preparation	Particle Size (nm)	Observations	Ref.
Carbamazepine–saccharin, indomethacin–saccharin, furosemide–caffeine	Two-step procedure; ^a^ nanonizing by wet milling with stabilizers.	<300 (suspensions)	Dissolution profiles improved compared to cocrystals and nanocrystals. Formation of nano-cocrystals suspensions required a rigid lattice and strong binding energy.	[106]
Myricetin–nicotinamide	One-step procedure ^b^ using top-down (grinding) and bottom-up (solution method with sonochemistry) approaches.	<1000	Both methods successfully generated nano-cocrystals, but the nano-cocrystal obtained by sonochemistry was more uniform. Improved dissolution rate for the nano-cocrystals prepared by the bottom-up approach in comparison with the nano-cocrystals prepared by grinding and the cocrystals.	[89]
Phenazopyridine–phthalimide	One-step procedure using a sonochemical approach.	21.4 ± 0.1 (diameter)	Greater in vitro dissolution rate and oral absorption in rats than the hydrochloride salt and cocrystals.	[97]
Paclitaxel (PTX)–disulfiram (DSF)	One-step procedure using antisolvent precipitation in presence of stabilizer (β-lactoglobulin).	160	Enhanced cytotoxicity toward taxol-resistant cells in comparison to free PTX-DSF formulation. Enhanced apoptosis in A549/TAX cells compared to free PTX-DSF formulation, improving the antitumor efficiency in vitro. Reduced IC_50_ (7-fold) and decreased dose (8.9-fold) compared to PTX.	[101]
Indomethacin–saccharin	One-step procedure using electrospray deposition.	219	Higher dissolution rate (3-fold) in comparison to the cocrystal prepared by solvent evaporation.	[107]
Baicalein–nicotinamide	Two-step procedure; nanonizing by high-pressure homogenization.	251.53	Improved in vitro dissolution rate in FaSSIF-V2 (2.17-fold) and FaSSGF (2.54-fold) in comparison to Baicalein coarse powder. Greater oral bioavailability in rats (6.02-fold) compared to Baicalein coarse suspension.	[98]
Ezetimibe–oxalic acid, ezetimibe–succinic acid, ezetimibe–maleic acid	One-step procedure using solvent evaporation and antisolvent precipitation.	226.4 ± 53 (ezetimibe-maleic acid nano-cocrystal by solvent evaporation)	Nano-cocrystals prepared with maleic acid as coformer showed higher solubility and dissolution than the nano-cocrystals prepared with oxalic acid and SUC, and pure ezetimibe.	[108]
Diclofenac–proline	Two-step procedure; nanonizing by top-down (wet milling and neat grinding) and bottom-up (globule inversion phase and fast evaporation assisted microwaving) approaches.	598.2 ± 63.2 (nano-cocrystals prepared by fast evaporation)	Superior dissolution and diffusion profile than the cocrystal.	[109]
Lamivudine (3TC)–zidovudine (AZT)	One-step procedure using bottom-up approaches (pseudo one-solvent cold-sonochemical precipitation with/without stabilizers).	<1000 (surfactant-coated nano-cocrystal)	Surfactants during sonication were necessary to produce surfactant-coated nano-cocrystal.	[105]
3TC-AZT (stabilized with SDS 0.90% *w*/*v* and TPGS 1000 1.40% *w*/*v*)	One-step procedure using bottom-up approaches (pseudo one-solvent cold-sonochemical precipitation).	332.9 ± 42.85	Improved cell viability (HeLa cells) in comparison to individual APIs and the 3TC-AZT physical mixture.	[100]
Carbamazepine–nicotinamide	One-step procedure using antisolvent precipitation with stabilizers.	D10 = 68.9 ± 9.5, D50 = 138.2 ± 16.6, D90 = 260.3 ± 17.96 (with 0.3% PVPVA-64)	Stabilizer type and its concentration affected the nano-cocrystal particle size.	[90]
4-Aminosalysilic acid–sulfamethazine (SUL)	One-step procedure using high-pressure homogenization (HPH) and high-power ultrasound (HPU).	HPH yielded nano-cocrystals, HPU micron-sized cocrystals	Nano-cocrystals showed a higher enhancement in dissolution rate; however, both the nano-cocrystals and micron-sized cocrystals enhanced the dissolution of SUL.	[110]
Carvedilol (CAR)–tartaric acid	One-step procedure using antisolvent precipitation (in presence of POL 188) with ultrasonication, followed by lyophilization.	0.98	Increased solubility (2000-fold) in comparison to pure CAR. Long-term physical stability for PEG protected slow-frozen nano-cocrystals.	[102]
3TC-AZT	Two-step procedure; nanonizing by top-down approach (wet media milling with surfactants).	271.0 ± 92.0	Improved cell viability (HeLa cells) in comparison to individual raw materials and the 3TC-AZT physical mixture. Statistically similar cell viability in comparison to the bottom-up nano-cocrystal.	[100]
3TC-AZT (redispersed in a stimuli-responsive carrier)	One-step procedure using bottom-up approach (cold-sonochemical precipitation with sodium lauryl sulfate and TPGS 1000) followed by redispersion in F-127 gel).	332.9 ± 42.85 (nano-cocrystal) 243.6 ± 26.58 (nano-cocrystal in gel)	Complete release of APIs from the nano-cocrystal-loaded-gel, but incomplete from the cocrystal-loaded gel and the physical mixture. Improved HeLa cell viability (88.0% ± 5.0%) for the nano-cocrystal-loaded-gel compared to the nano-cocrystal in aqueous media (76.9% ± 5.0%).	[111]
Itraconazole–fumaric acid, Itraconazole– succinic acid, indomethacin–saccharin, indomethacin–nicotinamide	Two-step procedure; nanonizing by wet milling.	300–450	Increased kinetic solubility and dissolution rate compared to nanocrystals and cocrystals.	[112]
(*S*)-Naproxen–nicotinamide	One-step procedure using surfactant-assisted grinding (with solutions of non-ionic surfactants or PEG 6000) and top-down wet milling.	<1000	Surfactant-assisted grinding generated the nano-cocrystal in a single-step process.	[113]
Cytarabine (ARA)–uracil (UR)	Two-step procedure; nanonizing by antisolvent precipitation.	562.70 ± 30.79	Diminished solubility but increased in vitro permeability. Enhanced antitumor activity in vitro compared to the ARA-UR cocrystal. Increased bioavailability and extended half-life in rats compared to pure ARA and the ARA-UR cocrystal.	[99]
Andrographolide (AG)–SA	Two-step procedure; nanonizing by hummer acoustic resonance.	190	Increased solubility in pH 1.2 HCl buffer (5.74 times) and pH 6.8 phosphate buffer (6.82 times) compared to raw AG.	[114]

^a^ Two-step procedure: the cocrystal is synthesized and isolated, and then nanonized. ^b^ One-step procedure: the API and coformer are paired and nanonized with/without stabilizer/s.

The improved properties of nano-cocrystals compared to cocrystals and multicomponent nanocrystals, the variety of available synthesis methods to achieve the desired outcome, and their broad applicability suggest that they will play a significant role in the pharmaceutical industry. However, unlike multicomponent nanocrystals and cocrystals, no nano-cocrystals have yet entered clinical development or been marketed. Various aspects and hurdles still need to be identified and overcome for translating pharmaceutical nano-cocrystals to the market. They include, among others, achieving the most suitable approaches for their formation [92]; to overcome production challenges [115]; stability issues (nano-cocrystals can experiment Ostwald ripening, agglomeration, polymorphic transformations, and cocrystal dissociation); uncertain commercial incentives; competition from simpler formulations (e.g., amorphous dispersions) or established nano drug-delivery systems [96]; and complex analytical characterization (it is difficult to simultaneously characterize cocrystal stoichiometry, nanostructure, surface chemistry, and physical and chemical stabilities). Another main hurdle, which is unique for nano-cocrystals, is regulatory uncertainty. In fact, neither the United States Food and Drug Administration (US FDA) nor the European Medicines Agency (EMA) has specific guidance or reflection papers addressing nano-cocrystals. For instance, the US FDA provides guidance documents on standard cocrystals [116] and on nanomaterial-containing products [117]; however, the latter guidance primarily covers nanoscale materials, including multicomponent nanocrystals, and does not explicitly address nano-cocrystals. In the case of the EMA, there is a reflection paper on cocrystals [118], various documents on nanomedicines, and a recent report on nanotechnology-based medicinal products [119]; however, none of these cover nano-cocrystals. The development of a clear regulatory framework and the overcoming of the various challenges associated with nano-cocrystals are expected to increase their importance as an innovative approach for optimizing API properties, reducing doses and side effects, and targeting various diseases in the near future.

### 3.3. Other Innovative Platforms

Microneedles have emerged as one of the most innovative and rapidly advancing platforms in drug delivery, offering a minimally invasive alternative capable of bridging the gap between traditional transdermal patches and hypodermic injections. Microneedles consist of micro-scale projections arranged on a supporting base, typically ranging from 25 to 2500 µm in length. These dimensions allow penetration of the stratum corneum while avoiding contact with deeper dermal nerves and vasculatures. This unique architecture enables painless or near-painless administration, facilitates self-application, and reduces risks associated with conventional injections, including needle phobia, needlestick injuries, and requirements for trained personnel [120,121,122,123].

Although microneedle technology has matured considerably, with substantial advances in fabrication precision and device consistency, several challenges still limit its broader clinical translation. Key issues include maintaining mechanical integrity during insertion, achieving adequate drug loading capacity, and ensuring the stability of incorporated therapeutics throughout processing and storage [124,125]. These limitations are particularly restrictive for APIs with unfavorable physicochemical properties, precisely the type of compounds that may benefit most from solid-state modulation strategies such as pharmaceutical cocrystals [20,126].

In this context, the recent study by Fandiño et al. [127] represents a significant milestone as it constitutes the first reported integration of a pharmaceutical cocrystal into a microneedle-based system. The authors developed a dissolving microneedle array incorporating an isoniazid cocrystal with lower solubility than the parent drug, designed to provide long-acting release. Their findings demonstrated that the cocrystal could be successfully embedded within the microneedle matrix without compromising the mechanical properties required for effective skin insertion. Moreover, the modified solid form enabled a controlled-release profile consistent with prolonged therapeutic exposure, highlighting the synergy between cocrystal engineering and microneedle delivery. The study also showed that combining these two technologies can overcome the formulation barriers typically associated with isoniazid, offering a promising strategy to improve adherence to long-term treatments.

## 4. Cocrystal Screening by Artificial Intelligence

Recently, artificial intelligence (AI) has significantly transformed the scientific method, emerging as a promising tool for generating predictive models that provide alternatives to tedious experimental work. In the field of pharmaceutical cocrystals, AI-driven models accelerate their screening at early stages of development using fundamental principles of machine learning algorithms [128,129,130,131,132]. Despite these advances, the inherent complexity of designing and implementing such algorithms continues to limit their widespread application. Among the pioneering contributions of AI in the cocrystal field are studies focused on determining the probability of cocrystallization for particular molecular pairs, aiming to encode key molecular descriptors related to functional groups, molecular size, and shape of the coformers. In addition, more recent studies have extended AI-based approaches toward cocrystal property prediction with relevant targeted physicochemical properties parameters, such as the lattice energy, density, melting temperature, crystal density, enthalpy and entropy of melting, as well as the ideal solubility of cocrystals. These predictive efforts emphasize the increasing potential of AI as a supplementary tool to rational cocrystal design, enabling more efficient selection of coformers and reducing experimental trial-and-error.

To expand the diversity of available datasets for APIs and coformers, data-driven models can be explored in combination with quantum chemical calculations. In this context, quantum data can be used as inputs to data-driven models such as machine learning models. Therefore, hybrid models can be built considering the molecular structure of compounds for screening pharmaceutical cocrystals. The effectiveness of such approaches strongly depends on selecting models tailored to the specific prediction task, according to data availability and target outputs. In this regard, the proposed models and optimization strategies represent a novel contribution, as they are specifically developed for the analysis and screening of pharmaceutical cocrystal coformers using molecular descriptors as input variables. For example, Alharby et al. [132] developed a machine learning-based framework to predict Hansen solubility parameters in the preparation of pharmaceutical cocrystals with improved properties. In another approach, Gubina et al. [133] reported an automated cocrystal design strategy aimed at improving tabletability profiles, using a novel screening pipeline that integrates deep generative models combined with optimization algorithms for extensive and systematic exploration of the target chemical space of potential coformers.

In addition, several computational approaches have been reported for prediction of cocrystal formation. For example, Devogelar et al. [134] introduced a data-driven methodology for the prediction of binary cocrystal formation using two types of artificial neural network models and cocrystal data extracted from the Cambridge Structural Database. Similarly, Mswahili et al. [130] also developed a predictive model for the formation of cocrystals of API molecules, comparing two feature selection algorithms. They demonstrated that the artificial neural network model achieved the best performance of all the known machine learning models. More recently, Yang et al. [131] proposed a cocrystal prediction method based on the XGBoost machine learning model. This method provides an efficient and accurate approach to the rational design and high-throughput screening of potential cocrystal systems.

## 5. Patents

When pharmaceutical cocrystals, as well as their formulations, demonstrate promising outcomes, several aspects of their intellectual property may be under protection to safeguard innovation. To qualify for patent protection, they must satisfy the requirements of novelty, non-obviousness, and utility or usefulness [135,136,137]. Over the last decade, several patents with academic or commercial interest have been approved, including cocrystals, multidrug cocrystals, and pharmaceutical cocrystal formulations.

Several patented inventions from Theapin Pharmaceuticals Inc. report the development of water-soluble cocrystal-based formulations with potential therapeutic applications. One patent describes an intravenous formulation containing acetylsalicylic acid and theanine cocrystals, which exhibit enhanced stability and bioactivity and can be used to treat patients with acute myocardial infarction [138]. Another invention described a sublingual formulation containing water-soluble cocrystals of acetylsalicylic acid combined with citric acid, sodium bicarbonate, and L-theanine, also intended for the treatment of acute myocardial infarction [139]. Additionally, a soluble cocrystal antioxidant formed by protocatechuic acid and L-theanine has been developed, which can be administered through different routes for the treatment of oxidative stress and inflammatory conditions [140].

Another invention relates to cocrystals of (rac)-tramadol and celecoxib for the treatment of moderate-to-severe [141]. Patent CN111205332A [142] describes the oxaliplatin–flavonoids (baicalein and naringenin) cocrystals that exhibit enhanced solubility and stability in the gastrointestinal tract, while reducing side effects and toxicicity, hence improving the therapeutic activity against human gastric adenocarcinoma cells. In addition, the Patent WO/2021/022103A1 [143] relates to cocrystals of posaconazole and a coformer comprising a selected functional group consisting of carboxyl, hydroxyl, carbonyl, amine, amide, and nitro groups, or any combination thereof. Methods of synthesis of the cocrystals are also provided, as well as the pharmaceutical compositions for oral administration, which may be formulated as tablets, capsules, granules, powders, or suspensions, and for parenteral administration, they may be formulated as injections (intravenous, intramuscular, or subcutaneous), drop infusion preparations, or suppositories. These cocrystals or their pharmaceutical compositions may be utilized to treat or prevent fungal, yeast, and/or dermatophyte infections. For instance, they can be employed for blastomycosis, aspergillosis, histoplasmosis, onychomycosis, coccidioidomycosis, Para coccidioidomycosis, cryptococcosis, mucormycosis, dermatophyte, and/or candidiasis infections. Another Patent, EP3674287A1 [144], relates to novel cocrystals combining R-baclofen with cinnamic acid, benzoic acid, salicylic acid, and ferulic acid, which have improved properties. The patent also refers to the manufacturing process and to pharmaceutical compositions containing aforementioned cocrystals, as well as to their use in solid dosage forms for oral administration. These dosage forms can be utilized to treat diseases or disorders such as spasticity due to multiple sclerosis, spinal cord injury, or cerebral palsy, as well as alcoholism.

An overview of cannabinoid pharmaceutical developments provides evidence of patents for cannabinoid cocrystals for therapeutic or cosmetic use. For example, Patent WO2020089424A1 [145] relates to a suitable solid composition for the oral administration of cannabinoids using one or more cocrystals of non-crystalline cannabinoids or cannabinoid cocrystals. This invention overcomes the chemical instability of cannabinoids during manufacturing and storage, as well as their low bioavailability and the side effects associated with oral administration. Another Patent, EP4085041A1 [146], describes cocrystals of cannabinol and tetrahydrocannabinol with a coformer (tetramethylpyrazine, L-proline, or D-proline) and the methods of preparing these cocrystals from a cannabinoid oil. This patent suggests that these cocrystals could be used as analgesics and/or antibiotics, or to treat diseases that respond to the immunosuppressive and anti-inflammatory properties of cannabinoids. These diseases include emesis, pain, epilepsy, Alzheimer’s disease, Huntington’s disease, Tourette’s syndrome, glaucoma, osteoporosis, schizophrenia, cancer, obesity, autoimmune diseases, diabetic complications, infections caused by methicillin-resistant *Staphylococcus aureus*, nausea, depression, anxiety, hypoxia–ischemia injuries, psychosis, and inflammatory diseases. In addition, Patent US20240315937A1A [147] describes a topical water-based formulation comprising a cannabinoid material for cosmetic applications. This invention provides cosmetic benefits such as moisturizing, soothing, antioxidant, anti-inflammatory, and anti-aging effects. Furthermore, it improves stability, epidermal delivery, and overall skin protective effects.

## 6. Future Perspectives

In the coming years, the incorporation of pharmaceutical cocrystals into practical and scalable drug-delivery platforms is expected to drive the most significant advances in cocrystal research. Cocrystal engineering will continue to emerge as a rational and versatile strategy to overcome key barriers, including the poor solubility, permeability, or stability of many APIs. This approach will enable the optimization of API properties and will facilitate the integration of cocrystals into advanced pharmaceutical delivery systems.

Several challenges must be carefully addressed to translate innovative cocrystal-based technologies from laboratory research to clinical practice. These challenges will include the development of reliable and predictive methods for cocrystals screening, the scalability and reproducibility of manufacturing processes, and complete solid-state characterization to guarantee phase purity, stability, and consistency throughout the product lifecycle. In addition, pharmaceutical evaluation will remain critical, as formulation-dependent behavior, potential phase transformation during processing or dissolution, and the influence of excipients can significantly impact in vivo performance.

Despite these challenges, the expanding clinical use of cocrystal-based systems across multiple routes of administration is expected to be highly promising. Intranasal formulations incorporating cocrystals are anticipated to span a wide range of therapeutic areas, from analgesics and antimigraine agents to emergency anticonvulsants and central nervous system-active peptides. Furthermore, the incorporation of cocrystals into microneedle-based delivery systems is expected to offer significant therapeutic potential for transdermal administration. As cocrystal science matures, research efforts are likely to increasingly focus on device-compatible manufacturing, nanoscale engineering, and the development of multicomponent API systems capable of translating molecular-level advantages into meaningful clinical and industrial impacts.

## 7. Conclusions

Over the past decade, solid-state formulations within the field of crystal engineering have advanced considerably, demonstrating how the deliberate manipulation of molecular assemblies through cocrystallization can effectively overcome unfavorable physicochemical properties of many APIs. Significant advances have been achieved in cocrystal synthesis methods, rational coformer selection based on property-driven design, and the adaptability of these strategies to different routes of administration, thereby expanding the practical relevance of pharmaceutical cocrystals.

As highlighted throughout this review, the field has progressively evolved from fundamental laboratory research toward industrial translation, a transition reflected in the growing number of patents and intellectual property filings. This advanced stage of development is characterized not only by the refinement of cocrystal design strategies, but also by the integration of solid-state knowledge into innovative drug-delivery platforms, such as 3D printing, nano-cocrystals, and even microneedle-based systems. Furthermore, the growing demand for novel cocrystal development has stimulated the adoption of artificial intelligence in pharmaceutical cocrystallization, offering significant opportunities to accelerate the discovery, screening, and development of cocrystals. Collectively, these advances illustrate the versatility of pharmaceutical cocrystals as modular tools capable of bridging supramolecular design with modern pharmaceutical delivery technologies.

Nevertheless, the path toward commercialization for certain innovative platforms, particularly nano-crystal-based systems, remains complicated due to regulatory frameworks still evolving and currently lacking specific guidance. Continued multidisciplinary collaboration among academia, industry, and regulatory agencies will be crucial to address these issues and to optimize the translational potential of pharmaceutical cocrystals in clinically relevant drug-delivery applications.

## Figures and Tables

**Table 1:** Summary of traditional cocrystal-based formulations.

Administration Route	Cocrystal	Method of Preparation	Observations	Ref.
Oral	Ketoconazole–fumaric acid	Controlled cocrystallization process by cooling	Bioavailability improved by increased cocrystal solubility and fumaric acid-mediated controlled release of ketoconazole.	[21]
Oral	Curcumin–*N*-acetylcysteine	Antisolvent gas technique using supercritical carbon dioxide	Enhanced antinociceptive and anti-inflammatory effects, related to improved bioavailability.	[22]
Pulmonary	Remdesivir– salicylic acid	Combined liquid-assisted grinding and thermal annealing	Cocrystal successfully reproduced using spray drying for inhaled dry powder formulation.	[23]
Intranasal	Favipiravir– isonicotinamide	Neat grinding combined with spray freeze-drying for intranasal dry powder formulation	Enhanced adhesion to nasal mucosa and prolonged retention time to nasal epithelium.	[24]
Transdermal	Meloxicam–salicylic acid	Crystallization method at room temperature	Reduced permeation by incorporation on non-ionic gel due to higher viscosity in comparison to the suspension.	[25]
Topical	Curcumin–pyrogallol	Liquid-assisted grinding method	Cocrystal incorporated into ointment accelerated complete skin regeneration and minimized scar formation.	[26]

## Data Availability

Not applicable.

## Abbreviations

The following abbreviations are used in this manuscript:

3TC	Lamivudine
AG	Andrographolide
API	Active pharmaceutical ingredient
ARA	Cytarabine
AUC	Area Under the Curve
AZT	Zidovudine
Be	Betaine
CAR	Carvedilol
CBZ	Carbamazepine
CNS	Central Nervous System
CTC	Tramadol–celecoxib cocrystal
DSF	Disulfiram
EMA	European Medicines Agency
FAV	Favipiravir
FDA	Food and Drug Administration
FFA	Flufenamic acid
HPH	High-Pressure Homogenization
HPU	High-Power Ultrasound
INA	Isonicotinamide
NSAID	Nonsteroidal Anti-Inflammatory Drug
PIC	Picolinamide
PTX	Paclitaxel
RDV	Remdesivir
SA	Salicylic acid
SUC	Succinic acid
SUL	4-Aminosalysilic acid-sulfamethazine
US	United States
